# Adaptive Super-Twisting Sliding Mode Control for Robot Manipulators with Input Saturation

**DOI:** 10.3390/s24092783

**Published:** 2024-04-26

**Authors:** Chenghu Jing, Hui Zhang, Yafeng Liu, Jing Zhang

**Affiliations:** 1 Henan Key Laboratory of Superhard Abrasives and Grinding Equipment, Henan University of Technology, Zhengzhou 450001, China; 2 School of Mechanical and Electronic Engineering, Henan University of Technology, Zhengzhou 450001, China; yfliu@haut.edu.cn; 3 School of Electrical Engineering and Automation, Henan University of Technology, Zhengzhou 450001, China; huizh2021@haut.edu.cn; 4 National Wuhu Robot Industry Achievement Transformation Center, Wuhu 241000, China; 2220910113@stu.ahpu.edu.cn

**Keywords:** robot manipulators, robust adaptive, super twisting, sliding mode, input saturation, finite time

## Abstract

The paper investigates a modified adaptive super-twisting sliding mode control (ASTSMC) for robotic manipulators with input saturation. To avoid singular perturbation while increasing the convergence rate, a modified sliding mode surface (SMS) is developed in this method. Using the proposed SMS, an ASTSMC is developed for robot manipulators, which not only achieves strong robustness but also ensures finite-time convergence. The boundary of lumped uncertainties cannot be easily obtained. A modified adaptive law is developed such that the boundaries of time-varying disturbance and its derivative are not required. Considering input saturation in practical cases, an ASTSMC with saturation compensation is proposed to reduce the effect of input saturation on tracking performances of robot manipulators. The finite-time convergence of the proposed scheme is analyzed. Through comparative simulations against two other sliding mode control schemes, the proposed method has been validated to possess strong adaptability, effectively adjusting control gains; simultaneously, it demonstrates robustness against disturbances and uncertainties.

## 1. Introduction

Robot manipulators have extensive application in various fields, such as the manufacturing industry [1], sorting systems [2], quadruped robots [3], and rehabilitation exoskeletons [4]. Recently, its trajectory-tracking control has received significant attention from researchers. Because of its easy implementation in practice, proportional-integral-derivative (PID) control was used for robot manipulators [5]. However, PID control cannot make dynamic systems achieve the required performances when the required performances are high, or the operating conditions often vary. Designing a high-performance trajectory-tracking control of robot manipulators is challenging due to their highly coupled and nonlinear features [6]. In addition, nonlinear friction, parameter variations, unmodelled dynamics, payload variations, and external disturbances always exist in the robotic system [7,8], which adversely affect the desired control performances. To achieve the good performance of robot manipulators under different operating conditions, there is no doubt that advanced control schemes insensitive to various disturbances are absolutely necessary. Various advanced approaches such as computed torque control [9], robust disturbance-rejection control [10], model predictive control [11], robust adaptive control [12], intelligent control method [13], sliding mode control (SMC) [14], and so on were investigated for the control of robotic manipulators.

Recently, SMC has been widely studied for various systems with uncertainties and external disturbances because of its strong robustness against various disturbances, guaranteed stability, fast response, and reasonable computational simplicity [15]. In [16,17], SMC was studied for robot manipulators. In [18,19], the adaptive law was introduced into SMC to estimate unknown parameters or gains of robot manipulators, which could avoid choosing large coefficients of the switching term. However, SMC adopts linear SMS on which the state variables usually converge to equilibrium points asymptotically as time tends to infinity. To increase the convergence rate, terminal SMC (TSMC) was studied for robot manipulators [20]. However, TSMC has a smaller convergence rate when state variables are far from equilibrium points. The fast TSMC (FTSMC) was proposed for robot manipulators to achieve fast transient convergence whether state variables are near or far from equilibriums [21]. It can be found that there exists a singularity problem in both TSMC and FTSMC [22]. Nonsingular TSMC (NTSMC) was proposed to solve the problem. In [23], a global NTSMC was proposed for n-link rigid manipulators to achieve finite time convergence. In the work, a terminal SMS was proposed to avoid singularity. In [24], an adaptive nonsingular FTSMC was presented for uncertain dynamic systems. In this control scheme, an adaptive parameter-tuning approach was used for the unknown bounds of uncertainties, such that the boundary of the uncertainties and disturbances was not required in advance. Then, this control scheme was applied to robot manipulators to verify its effectiveness. The discontinuous term could cause the chattering. The boundary layer approach is one of the common methods to reduce the chattering. However, it loses the finite time convergence in the boundary layer. In [25], a continuous TSMC scheme for robot manipulators is proposed. A fast continuous reaching law was used instead of discontinuous reaching law for chattering-free. However, the errors could not converge to zero in finite time owing to the bounded uncertainties. In [26], Mondal addressed an adaptive second-order TSMC for robot manipulators in which an adaptive method was used to obtain the estimation of the bound of disturbances. In this technique, the derivative of the control law is designed according to the framework of TSMC. Then, the final control law was obtained after integration, which makes the control law continuous. This control technique is chattering-free. Second-order SMC (SOSMC) is the most common approach among high-order SMC that possesses robustness against disturbance and uncertainty and alleviates the chattering phenomenon if appropriately used [27].

For SOSMC, the super-twisting algorithm (STA) is a promising technology. It only requires the measurement of SMS [28]. A lot of works on STA have been conducted [29,30,31,32,33]. Kali developed an SMC scheme based on the time-delay estimation for the control of uncertain robotic manipulators [34]. In this presented controller, the time-delay estimation was applied to obtain an estimation of uncertainties, and standard STA was designed to eliminate estimation errors and strengthen system robustness. In [35], a robust super-twisting SMC (STSMC) of robotic systems was developed. This control approach adopted prescribed performance control to ensure the tracking performance of robotic systems. However, the two approaches required that the upper bound of the disturbance’s derivative could be obtained. An adaptive control based on global STSMC was proposed for n-link robot manipulators in [36]. However, the authors only analyzed the stability of adaptive global sliding mode control, while stability analysis of adaptive global STSMC was not presented.

In the above-mentioned works, the input saturation constraint of robot manipulators is not considered. Some works were conducted to eliminate the effect of input saturation on control systems [37,38]. This work will investigate the adaptive STSMC for the finite-time tracking control of robot manipulators with input saturation. In comparison with the previous works, the main contributions of the work are demonstrated as:

(1) A novel SMS is proposed to obtain fast convergence and avoid singular problems.
(2) An ASTSMC for robot manipulators is developed and analyzed, which could achieve finite-time convergence, strong robustness, good adaptability, and high accuracy.
(3) An ASTSMC with saturation compensation (ASTSMCSC) is presented, which could improve the tracking performances of robot manipulators with input saturation.

## 2. Dynamic Model

The dynamics of a series multiple-joint robot manipulator are expressed by

(1) $$M_{0}\left(q\right)\ddot{q}+C_{0}\left(q,\dot{q}\right)\dot{q}+G_{0}\left(q\right)=\tau +\Delta ,$$

where $M_{0}\left(q\right)$ denotes the inertia matrix, $q={\left[q_{1},q_{2},\cdots ,q_{n}\right]}^{T}$ is the angular position, τ is the control torque, $C_{0}\left(q,\dot{q}\right)$ denotes the Coriolis-centrifugal matrix, Δ denotes the disturbances, and $G_{0}\left(q\right)$ denotes the gravitational vector. Here, n denotes the number of joints of robot manipulators.

**Remark** **1.**  *$M_{0}\left(q\right)$, $C_{0}\left(q,\dot{q}\right)$ and $G_{0}\left(q\right)$ denote the nominal values. The uncertainties are integrated into the disturbance Δ.*

**Assumption** **1**  **([26,34]).** *The disturbance Δ and its derivative are bounded.*

## 3. Adaptive Super-Twisting Sliding Mode Control

For robotic manipulators, the objective of trajectory tracking control is to make the trajectory q track the reference trajectory $q_{d}$. Let $e=q-q_{d}$ denote the tracking error. A novel fast SMS is presented as

(2) $$\begin{array}{l} s=\dot{e}+\hbar_{1}e+℘\left(e\right)\\ ℘\left(e\right)=\hbar_{2}\mu \left(e\right){\mathrm{sig}}^{\alpha }\left(e\right) \end{array},$$

where $\hbar_{1}=\mathrm{diag}\left\{\hbar_{11},\hbar_{12},\cdots ,\hbar_{1n}\right\}$ and $\hbar_{2}=\mathrm{diag}\left\{\hbar_{21},\hbar_{22},\cdots ,\hbar_{2n}\right\}$ are diagonal matrices with strictly positive real elements, 0<α<1 is a constant, $s={\left[s_{1},s_{2},\cdots ,s_{n}\right]}^{T}$, ${\mathrm{sig}}^{\alpha }\left(e\right)={\left[{\left|e_{1}\right|}^{\alpha }\mathrm{sign}\left(e_{1}\right),\cdots ,{\left|e_{n}\right|}^{\alpha }\mathrm{sign}\left(e_{n}\right)\right]}^{T}$, $\mu \left(e\right)=\mathrm{diag}\left\{\mu \left(e_{1}\right),\cdots ,\mu \left(e_{n}\right)\right\}\in R^{n\times n}$. Here, $\mu \left(e_{i}\right)$ is defined as

(3) $$\mu \left(e_{i}\right)=\{\begin{array}{cc} 1 & ,{\left|e_{i}\right|}^{1-\alpha }\geq \theta_{i}\\ \sin\left(\frac{\pi }{2}\frac{{\left|e_{i}\right|}^{1-\alpha }}{\theta_{i}}\right) & ,{\left|e_{i}\right|}^{1-\alpha }<\theta_{i} \end{array},$$

where $\theta_{i}$ is a small and positive design parameter.

Reorganize Equation (1) as

(4) $$\ddot{q}=M_{0}^{-1}\left(q\right)\left(\tau -C_{0}\left(q,\dot{q}\right)\dot{q}-G_{0}\left(q\right)\right)+f_{d},$$

where $f_{d}=M_{0}^{-1}\left(q\right)\Delta$.

**Assumption** **2.**  *The disturbance $f_{d}$ is continuously changing, i.e., there exists a constant $\gamma_{i2}>0$ such that $\left|{\dot{f}}_{di}\right|\leq \gamma_{i2}$, where ${\dot{f}}_{di}$ represents the i-th element of the vector ${\dot{f}}_{d}$.*

**Remark** **2.**  *In many real-world mechanical systems, disturbances typically evolve smoothly over time rather than manifesting as instantaneous jumps, except perhaps in instances influenced by extreme events or noise. Therefore, this paper does not consider sudden disturbance.*

Using Equations (2) and (4), one obtains

(5) $$\begin{array}{l} \dot{s}=\ddot{e}+\hbar_{1}\dot{e}+\dot{℘}\left(e\right)\\ =\ddot{q}-{\ddot{q}}_{d}+\hbar_{1}\dot{e}+\dot{℘}\left(e\right)\\ =M_{0}^{-1}\left(q\right)\left(\tau -C_{0}\left(q,\dot{q}\right)\dot{q}-G_{0}\left(q\right)\right)+f_{d}-{\ddot{q}}_{d}+\hbar_{1}\dot{e}+\dot{℘}\left(e\right) \end{array},$$

where $\dot{℘}\left(e\right)$ denotes the derivative of ℘(e), and its element $\dot{℘}\left(e_{i}\right)$ is provided by

(6) $$\dot{℘}\left(e_{i}\right)=\{\begin{array}{ll} \alpha \hbar_{2i}{\left|e_{i}\right|}^{\alpha -1}{\dot{e}}_{i} & ,\;{\left|e_{i}\right|}^{1-\alpha }\geq \theta_{i}\\ \alpha \hbar_{2i}\sin\left(\frac{\pi }{2}\frac{{\left|e_{i}\right|}^{1-\alpha }}{\theta_{i}}\right){\left|e_{i}\right|}^{\alpha -1}{\dot{e}}_{i}+\cos\left(\frac{\pi }{2}\frac{{\left|e_{i}\right|}^{1-\alpha }}{\theta_{i}}\right)\frac{\hbar_{2i}\pi \left(1-\alpha \right)}{2\theta_{i}}{\dot{e}}_{i} & ,\;{\left|e_{i}\right|}^{1-\alpha }<\theta_{i}\;\mathrm{and}\;e_{i}\neq 0\\ \frac{\hbar_{2i}\pi }{2\theta_{i}}{\dot{e}}_{i} & ,\;e_{i}=0 \end{array},$$

Inspired by the work [29], according to Equation (5), a modified ASTSMC is developed as

(7) $$\begin{array}{cc} \tau & =C_{0}\left(q,\dot{q}\right)\dot{q}+G_{0}\left(q\right)+M_{0}\left(q\right){\ddot{q}}_{d}-M_{0}\left(q\right)\left(\hbar_{1}\dot{e}+\dot{℘}\left(e\right)\right)\\ & +M_{0}\left(q\right)\left(-k_{1}\sqrt{L}{\mathrm{sig}}^{\frac{1}{2}}\left(s\right)-k_{2}Ls-\frac{\dot{L}}{L}s+\delta \right)\\ \dot{\delta } & =-k_{3}L\mathrm{sign}\left(s\right)-k_{4}L^{2}s \end{array},$$

where $k_{1}=\mathrm{diag}\left\{k_{11},\cdots ,k_{1n}\right\}$, $k_{2}=\mathrm{diag}\left\{k_{21},\cdots ,k_{2n}\right\}$, $k_{3}=\mathrm{diag}\left\{k_{31},\cdots ,k_{3n}\right\}$ and $k_{4}=\mathrm{diag}\left\{k_{41},\cdots ,k_{4n}\right\}$ are control gains, and L is an adaptive coefficient, ${\mathrm{sig}}^{\frac{1}{2}}\left(s\right)={\left[{\left|s_{1}\right|}^{\frac{1}{2}}\mathrm{sign}\left(s_{1}\right),\cdots ,{\left|s_{n}\right|}^{\frac{1}{2}}\mathrm{sign}\left(s_{n}\right)\right]}^{T}$, and $\mathrm{sign}\left(s\right)={\left[\mathrm{sign}\left(s_{1}\right),\cdots ,\mathrm{sign}\left(s_{n}\right)\right]}^{T}$. For the adaptive coefficient L, the adaptive law is designed as

(8) $$\dot{L}=\{\begin{array}{cc} r\mathrm{sign}\left(\left\|s\right\|-\sigma \right) & ,\;L\geq L_{\min}\\ \kappa & ,\;L<L_{\min} \end{array},$$

where r, σ, κ, and $L_{\min}$ are positive constants.

**Remark** **3.**  *In comparison with the work [29], the modified ASTSMC (7) possesses two significant features. On the one hand, an additional item $\dot{L}s/L$ is added to the control law. On the other hand, adaptive law is modified and improved: (1) adaptive rate can be automatically adjusted in terms of sliding mode variable, rather than a constant; (2) a dead zone is introduced into adaptive law to avoid some effects of noise, discretization, and imperfections in the application; (3) the minimum value $L_{\min}$ is introduced to prevent the adaptive coefficient from becoming too small.*

In view of Equations (5) and (7), one obtains

(9) $$\dot{s}=-k_{1}\sqrt{L}{\mathrm{sig}}^{\frac{1}{2}}\left(s\right)-k_{2}Ls-\frac{\dot{L}}{L}s+\delta +f_{d},$$

Defining a new vector $s_{I}=\delta +f_{d}$, the dynamic system (9) is expressed as

(10) $$\begin{array}{l} \dot{s}=-k_{1}\sqrt{L}{\mathrm{sig}}^{\frac{1}{2}}\left(s\right)-k_{2}Ls-\frac{\dot{L}}{L}s+s_{I}\\ {\dot{s}}_{I}=-k_{3}L\mathrm{sign}\left(s\right)-k_{4}L^{2}s+{\dot{f}}_{d} \end{array},$$

**Theorem** **1.**  *Considering the model (4), the controller (7) with adaptive law (8) guarantees that a practical sliding-mode domain, i.e., $\left|s_{i}\right|\leq \delta_{i1}$ could be established if the control gains $k_{1i}$, $k_{2i}$, $k_{3i}$, and $k_{4i}$ are properly selected such that*

(11) $$9k_{1}^{2}k_{2}^{2}+8k_{2}^{2}k_{3}<4k_{3}k_{4},$$

**Proof** **of** **Theorem** **1.**  The dynamic system (10) is expressed as

(12) $$\begin{array}{l} {\dot{s}}_{i}=-k_{1i}\sqrt{L}{\mathrm{sig}}^{\frac{1}{2}}\left(s_{i}\right)-k_{2i}Ls_{i}-\frac{\dot{L}}{L}s_{i}+s_{Ii}\\ {\dot{s}}_{Ii}=-k_{3i}L\mathrm{sign}\left(s_{i}\right)-k_{4i}L^{2}s_{i}+{\dot{f}}_{di} \end{array},$$

For the convenience of proof, an auxiliary vector is defined as

(13) $$\eta ={\left[\eta_{1},\eta_{2},\eta_{3}\right]}^{T}={\left[\sqrt{L}{\mathrm{sig}}^{\frac{1}{2}}\left(s_{i}\right),Ls_{i},s_{Ii}\right]}^{T},$$

Thus, the system (12) is rewritten as

(14) $$\dot{\eta }=-\frac{L}{2\left|\eta_{1}\right|}\left[\begin{array}{ccc} k_{1i} & 0 & -1\\ 0 & 2k_{1i} & 0\\ 2k_{3i} & 0 & 0 \end{array}\right]\eta -L\left[\begin{array}{ccc} 0.5k_{2i} & 0 & 0\\ 0 & k_{2i} & 1\\ 0 & k_{4i} & 0 \end{array}\right]\eta +\left[\begin{array}{c} 0\\ 0\\ {\dot{f}}_{di} \end{array}\right],$$

Next, we need to prove that the vector η converges to a bounded domain. A Lyapunov function is considered as

(15) $$V_{1}=\frac{1}{2}\eta^{T}P_{1}\eta ,$$

where $P_{1}$ is a positive definite symmetric matrix, and it is written as

(16) $$P_{1}=\left[\begin{array}{ccc} 4k_{3}+k_{1}^{2} & k_{1}k_{2} & -k_{1}\\ k_{1}k_{2} & 2k_{4}+k_{2}^{2} & -k_{2}\\ -k_{1} & -k_{2} & 2 \end{array}\right],$$

From the definition (13), it follows that $V_{1}$ is a function of $s_{i}$ and $s_{Ii}$. the Lyapunov function $V_{1}$ in Equation (15) is everywhere continuous in the set ${ℜ}^{1}=\left\{\left(s_{i},s_{Ii}\right)\in R^{2n}\right\}$. A set is defined as ${ℜ}_{1}=\left\{\left(s_{i},s_{Ii}\right)\in R^{2n}:s_{i}=0\right\}$. Then, $V_{1}$ is differentiable everywhere except in the set ${ℜ}_{1}$. It is not difficult to verify that $V_{1}$ is not only positive definite but also radially unbounded. According to previous works [30], the derivative of $V_{1}$ can be organized as

(17) $${\dot{V}}_{1}\leq -\frac{L}{2\left|\eta_{1}\right|}\eta^{T}\Omega_{1}\eta -L\eta^{T}\Lambda_{1}\eta +{\dot{f}}_{di}\eta^{T}\psi_{1},$$

where $\psi_{1}={\left[\begin{array}{ccc} -k_{1i} & -k_{2i} & 2 \end{array}\right]}^{T}$, $\Omega_{1}$ and $\Lambda_{1}$ are matrices, and they are calculated as

(18) $$\begin{array}{l} \Omega_{1}=k_{1i}\left[\begin{array}{ccc} k_{1i}^{2}+2k_{3i} & 0 & -k_{1i}\\ 0 & k_{2i}^{2}+2k_{4i} & -3k_{2i}\\ -k_{1i} & -3k_{2i} & 1 \end{array}\right]\\ \Lambda_{1}=k_{2i}\left[\begin{array}{ccc} 2k_{1i}^{2}+k_{3i} & 0 & 0\\ 0 & k_{2i}^{2}+k_{4i} & -k_{2i}\\ 0 & -k_{2i} & 1 \end{array}\right] \end{array},$$

To guarantee that $\Omega_{1}$ and $\Lambda_{1}$ are positive definite, the condition (11) can be obtained. That is to say, $\Omega_{1}$ and $\Lambda_{1}$ are positive definite if the control gains $k_{1i}$, $k_{2i}$, $k_{3i}$, and $k_{4i}$ are properly selected to satisfy the condition (11).

Recall the following inequalities

(19) $$\begin{array}{l} \lambda_{\min}\left(P_{1}\right){\left\|\eta \right\|}^{2}\leq \eta^{T}P_{1}\eta =2V_{1}\leq \lambda_{\max}\left(P_{1}\right){\left\|\eta \right\|}^{2}\\ \lambda_{\min}\left(\Omega_{1}\right){\left\|\eta \right\|}^{2}\leq \eta^{T}\Omega_{1}\eta \leq \lambda_{\max}\left(\Omega_{1}\right){\left\|\eta \right\|}^{2}\\ \lambda_{\min}\left(\Lambda_{1}\right){\left\|\eta \right\|}^{2}\leq \eta^{T}\Lambda_{1}\eta \leq \lambda_{\max}\left(\Lambda_{1}\right){\left\|\eta \right\|}^{2} \end{array},$$

From the inequality (19), it follows that

(20) $$\begin{array}{l} \frac{2\lambda_{\min}\left(\Omega_{1}\right)}{\lambda_{\max}\left(P_{1}\right)}V_{1}\leq \eta^{T}\Omega_{1}\eta \leq \frac{2\lambda_{\max}\left(\Omega_{1}\right)}{\lambda_{\min}\left(P_{1}\right)}V_{1}\\ \frac{2\lambda_{\min}\left(\Lambda_{1}\right)}{\lambda_{\max}\left(P_{1}\right)}V_{1}\leq \eta^{T}\Lambda_{1}\eta \leq \frac{2\lambda_{\max}\left(\Lambda_{1}\right)}{\lambda_{\min}\left(P_{1}\right)}V_{1}\\ \left|\eta_{1}\right|\leq \left\|\eta \right\|\leq \sqrt{\frac{2}{\lambda_{\min}\left(P_{1}\right)}}V_{1}^{\frac{1}{2}} \end{array},$$

Substituting the inequalities (19) and (20) into the inequality (17) yields

(21) $$\begin{array}{l} {\dot{V}}_{1}\leq -\frac{L}{2\sqrt{\frac{2}{\lambda_{\min}\left(P_{1}\right)}}V_{1}^{\frac{1}{2}}}\frac{2\lambda_{\min}\left(\Omega_{1}\right)}{\lambda_{\max}\left(P_{1}\right)}V_{1}-L\frac{2\lambda_{\min}\left(\Lambda_{1}\right)}{\lambda_{\max}\left(P_{1}\right)}V_{1}+\gamma_{i2}\left\|\psi_{1}\right\|\left\|\eta^{T}\right\|\\ \leq -L\frac{\sqrt{\lambda_{\min}\left(P_{1}\right)}\lambda_{\min}\left(\Omega_{1}\right)}{\sqrt{2}\lambda_{\max}\left(P_{1}\right)}V_{1}^{\frac{1}{2}}-L\frac{2\lambda_{\min}\left(\Lambda_{1}\right)}{\lambda_{\max}\left(P_{1}\right)}V_{1}+\gamma_{i2}\left\|\psi_{1}\right\|\sqrt{\frac{2}{\lambda_{\min}\left(P_{1}\right)}}V_{1}^{\frac{1}{2}}\\ =-\left(L\lambda_{11}-\lambda_{12}\right)V_{1}^{\frac{1}{2}}-L\lambda_{13}V_{1} \end{array},$$

where $\lambda_{11}=\frac{\sqrt{\lambda_{\min}\left(P_{1}\right)}\lambda_{\min}\left(\Omega_{1}\right)}{\sqrt{2}\lambda_{\max}\left(P_{1}\right)}$, $\lambda_{12}=\gamma_{i2}\left\|\psi_{1}\right\|\sqrt{\frac{2}{\lambda_{\min}\left(P_{1}\right)}}$, and $\lambda_{13}=\frac{2\lambda_{\min}\left(\Lambda_{1}\right)}{\lambda_{\max}\left(P_{1}\right)}$.

First, the adaptive law is not considered, i.e., L is a constant. Obviously, if control gains $k_{1i}$, $k_{2i}$, $k_{3i}$, $k_{4i}$, and L are properly selected to satisfy the constraint $L\lambda_{11}-\lambda_{12}>0$, it is concluded from Lemma 1 in [29] that the states of the dynamic system (12) converge to zero. However, $\lambda_{12}$ is unknown due to the unknown boundary $\gamma_{i2}$. Parameter tuning is extremely difficult.

Therefore, the adaptive law (8) is adopted. When $\left|s_{i}\right|>\sigma$ and $L\geq L_{\min}$, the adaptive law becomes $\dot{L}=r>0$. Then, the adaptive coefficient L starts to increase until the constraint $L\lambda_{11}-\lambda_{12}>0$ is met, which guarantees the finite time stability of the dynamic system (12). Then, $s_{i}$ converges to the domain $\left|s_{i}\right|\leq \delta_{i1}$. When $s_{i}$ converges to the domain $\left|s_{i}\right|\leq \sigma$, the adaptive law becomes $\dot{L}=-r<0$. The adaptive coefficient L starts to descend. When the coefficient L descends to a certain extent, the finite time stability is destroyed. Once the coefficient L is less than the predesigned minimum value $L_{\min}$, the adaptive law becomes $\dot{L}=\kappa >0$, which guarantees that the coefficient L starts immediately to increase. So, L must be larger than the parameter $L_{\min}$. Due to the decrease of the coefficient L, $\left|s_{i}\right|$ may become larger than the predesigned value σ. Then, the adaptive coefficient L starts again to increase until the stability of the dynamic system (12) is ensured. Therefore, it is concluded that $s_{i}$ always stays in larger regions where $\left|s_{i}\right|\leq \delta_{i1}$. □

**Remark** **4**  **([30,32]).** *The generalized Lyapunov theorem only requires continuity and not differentiability of the Lyapunov function $V_{1}$ along the solution trajectories. ${ℜ}_{1}^{\prime }=\left\{\left(s_{i},s_{Ii}\right)\in R^{2n}:s_{i}=0,s_{Ii}=0\right\}$ is an equilibrium of the differential Equation (12). From Equation (12), if $\left(s_{i},s_{Ii}\right)\in {ℜ}_{1}\backslash {ℜ}_{1}^{\prime }$, then $s_{i}=0$ and ${\dot{s}}_{i}=s_{Ii}\neq 0$. Therefore, at least one component $s_{i}$ will monotonically cross zero unless $\left(s_{i},s_{Ii}\right)$ stay in the set ${ℜ}_{1}^{\prime }$.*

**Theorem** **2.**  *When* $s_{i}=0$*, the tracking error* $e_{i}$ *converges into a bounded region* $W_{1}=\left\{e_{i}:{\left|e_{i}\right|}^{1-\alpha }\leq \theta_{i}\right\}$ *in finite time, and converges asymptotically to zero.*

**Proof** **of** **Theorem** **2.**  When $s_{i}=0$, from the sliding mode variable (2), one obtains

(22) $${\dot{e}}_{i}+\hbar_{1i}e_{i}+\hbar_{2i}\mu \left(e_{i}\right){\left|e_{i}\right|}^{\alpha }\mathrm{sign}\left(e_{i}\right)=0,$$

Consider the Lyapunov function

(23) $$V_{e}=\frac{1}{2}e_{i}^{T}e_{i},$$

Making use of Equation (22), the derivative of $V_{e}$ is expressed by

(24) $$\begin{array}{cc} {\dot{V}}_{e} & =e_{i}^{T}{\dot{e}}_{i}\\ & =e_{i}^{T}\left[-\hbar_{1i}e_{i}-\hbar_{2i}\mu \left(e_{i}\right){\left|e_{i}\right|}^{\alpha }\mathrm{sign}\left(e_{i}\right)\right]\\ & \leq -\hbar_{1i}e_{i}^{2}-\hbar_{2i}\mu \left(e_{i}\right){\left|e_{i}\right|}^{\alpha +1}\\ & =-2\hbar_{1i}V_{e}-2^{\frac{\alpha +1}{2}}\hbar_{2i}\mu \left(e_{i}\right)V_{e}^{\frac{\alpha +1}{2}} \end{array},$$

When ${\left|e_{i}\right|}^{1-\alpha }\geq \theta_{i}$, the Lyapunov function $V_{e}$ satisfies ${\dot{V}}_{e}\leq -2\hbar_{1i}V_{e}-2^{\frac{\alpha +1}{2}}\hbar_{2i}V_{e}^{\frac{\alpha +1}{2}}$. Therefore, the tracking error $e_{i}$ converges into a bounded region ${\left|e_{i}\right|}^{1-\alpha }\leq \theta_{i}$ in finite time. Evidently, the region could be very small by turning down the constant $\theta_{i}$. When ${\left|e_{i}\right|}^{1-\alpha }<\theta_{i}$ the Lyapunov function $V_{e}$ satisfies ${\dot{V}}_{e}\leq -2\hbar_{1i}V_{e}$, the Equation (22) is still asymptotically stable. So, the tracking error $e_{i}$ can asymptotically converge to zero. □

**Remark** **5.**  *The convergence of* $e_{i}$ *when the real SMS* $s_{i}=0$ *is analyzed. If the real sliding mode region* $W_{2}=\left\{s_{i}:\left|s_{i}\right|\leq \delta_{i1}\right\}$ *is established, the tracking error* $e_{i}$ *can converge into a small bounded region in finite time, but not zero.*

## 4. Adaptive Super-Twisting Control with Saturation Compensation

The control law (7) cannot be directly put into use due to the torque limitations. In fact, the control torque τ is subject to the constraint $\tau_{l}\leq \tau \leq \tau_{u}$, where $\tau_{l}$ and $\tau_{u}$ represent the upper and lower bounds of the input constraint, respectively. Thus, the actual control input τ could be defined as

(25) $$\tau =\{\begin{array}{ll} \tau_{u}, & \mathrm{if}\;\bar{\tau }>\tau_{u}\;\\ \bar{\tau }, & \mathrm{if}\;\tau_{l}\leq \bar{\tau }\leq \tau_{u}\\ \tau_{l}, & \mathrm{if}\;\bar{\tau }<\tau_{l} \end{array},$$

where $\bar{\tau }$ denotes the desired control law that is designed without considering input constraints. Once the input saturation occurs, the tracking error e will increase such that the system trajectories will be away from SMS, which ruins the control performance under the control law (7). To handle input saturation (25), an auxiliary dynamic system is designed as

(26) $$\dot{\chi }=-a\chi +\tilde{\tau },$$

where $\tilde{\tau }=\tau -\bar{\tau }$ denotes the error of control input due to input saturation, and $a=\mathrm{diag}\left\{a_{1},\cdots ,a_{n}\right\}\in R^{n\times n}$ is the coefficient diagonal matrix.

An auxiliary vector is defined as

(27) $$\bar{s}=s-\chi ,$$

Using the dynamic system (5), the dynamics of vector $\bar{s}$ is provided as

(28) $$\begin{array}{cc} \dot{\bar{s}} & =\dot{s}-\dot{\chi }\\ & =M_{0}^{-1}\left(q\right)\left(\tau -C_{0}\left(q,\dot{q}\right)\dot{q}-G_{0}\left(q\right)\right)+f_{d}-{\ddot{q}}_{d}+\hbar_{1}\dot{e}+\dot{℘}\left(e\right)-\dot{\chi } \end{array},$$

Considering the input constraint (25), the control provided in (7) is modified as

(29) $$\begin{array}{cc} \bar{\tau } & =C_{0}\left(q,\dot{q}\right)\dot{q}+G_{0}\left(q\right)+M_{0}\left(q\right){\ddot{q}}_{d}-M_{0}\left(q\right)\left(\hbar_{1}\dot{e}+\dot{℘}\left(e\right)\right)-M_{0}\left(q\right)a\chi \\ & +M_{0}\left(q\right)\left(-k_{1}\sqrt{\bar{L}}{\mathrm{sig}}^{\frac{1}{2}}\left(\bar{s}\right)-k_{2}\bar{L}\bar{s}-\frac{\dot{\bar{L}}}{\bar{L}}\bar{s}+\bar{\delta }\right)\\ \dot{\bar{\delta }} & =-k_{3}\bar{L}\mathrm{sign}\left(\bar{s}\right)-k_{4}{\bar{L}}^{2}\bar{s} \end{array},$$

with the modified adaptive law

(30) $$\dot{\bar{L}}=\{\begin{array}{cc} r\mathrm{sign}\left(\left\|\bar{s}\right\|-\sigma \right) & ,\;\bar{L}\geq L_{\min}\\ \kappa & ,\;\bar{L}<L_{\min} \end{array},$$

**Figure 1:**
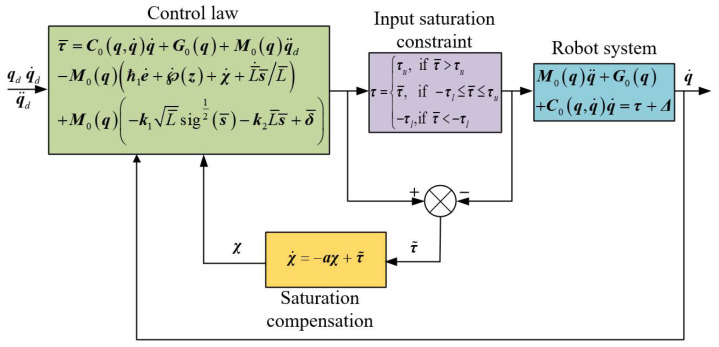
The proposed control architecture for robot systems with input saturation.

The architecture of the proposed ASTSMCSC is shown in Figure 1.

In view of the dynamic system (28) and control law (29), the dynamics of SMS are provided as

(31) $$\dot{\bar{s}}=-k_{1}\sqrt{\bar{L}}{\mathrm{sig}}^{\frac{1}{2}}\left(\bar{s}\right)-k_{2}\bar{L}\bar{s}-\frac{\dot{\bar{L}}}{\bar{L}}\bar{s}+\bar{\delta }+f_{d},$$

Defining an auxiliary vector ${\bar{s}}_{I}=\bar{\delta }+f_{d}$, the dynamic system (31) is expressed as

(32) $$\begin{array}{l} \dot{\bar{s}}=-k_{1}\sqrt{\bar{L}}{\mathrm{sig}}^{\frac{1}{2}}\left(\bar{s}\right)-k_{2}\bar{L}\bar{s}-\frac{\dot{\bar{L}}}{\bar{L}}\bar{s}+{\bar{s}}_{I}\\ {\dot{\bar{s}}}_{I}=-k_{3}\bar{L}\mathrm{sign}\left(\bar{s}\right)-k_{4}{\bar{L}}^{2}\bar{s}+{\dot{f}}_{d} \end{array},$$

The dynamic system (32) is expressed in scalar form as

(33) $$\begin{array}{l} {\dot{\bar{s}}}_{i}=-k_{1i}\sqrt{\bar{L}}{\mathrm{sig}}^{\frac{1}{2}}\left({\bar{s}}_{i}\right)-k_{2i}\bar{L}{\bar{s}}_{i}-\frac{\dot{\bar{L}}}{\bar{L}}{\bar{s}}_{i}+{\bar{s}}_{Ii}\\ {\dot{\bar{s}}}_{Ii}=-k_{3i}\bar{L}\mathrm{sign}\left({\bar{s}}_{i}\right)-k_{4i}{\bar{L}}^{2}{\bar{s}}_{i}+{\dot{f}}_{di} \end{array},$$

The principle framework of the control method proposed in this article is shown in Figure 1.

**Theorem** **3.**  *Considering the dynamic system (4) with the input saturation constraint (25) under Assumptions ~1–3, the control law (29) with the auxiliary dynamic system (26) and the adaptive law (30) can ensure the closed-loop system globally uniformly ultimately bounded, if the control gains* $k_{1i}$*,* $k_{2i}$*,* $k_{3i}$*, and* $k_{4i}$ *are properly selected to satisfy the condition (11).*

**Proof** **of** **Theorem** **3.**  For the convenience of proof, a vector is defined as

(34) $$\omega ={\left[\omega_{1},\omega_{2}\right]}^{T}={\left[\sqrt{\bar{L}}{\mathrm{sig}}^{\frac{1}{2}}\left({\bar{s}}_{i}\right),\bar{L}{\bar{s}}_{i},{\bar{s}}_{Ii}\right]}^{T},$$

Next, we need to prove that the vector converges to a bounded domain. To this end, a Lyapunov function is considered as

(35) $$V_{2}=\frac{1}{2}\omega^{T}P_{1}\omega ,$$

From the definition (34), it follows that $V_{2}$ is a function of ${\bar{s}}_{i}$ and ${\bar{s}}_{Ii}$. The Lyapunov function $V_{2}$ is everywhere continuous in the set ${ℜ}^{2}=\left\{\left({\bar{s}}_{i},{\bar{s}}_{Ii}\right)\in R^{2n}\right\}$. A set is defined as ${ℜ}_{2}=\left\{\left({\bar{s}}_{i},{\bar{s}}_{Ii}\right)\in R^{2n}:{\bar{s}}_{i}=0\right\}$. Then, the Lyapunov function $V_{2}$ is differentiable everywhere except in the set ${ℜ}_{2}$. It is not difficult to verify that the Lyapunov function $V_{2}$ is not only positive definite but also radially unbounded.

Using the primary analysis utilized in the proof of Theorem 1, the derivative of $V_{2}$ is provided by

(36) $${\dot{V}}_{2}\leq -\left(\bar{L}\lambda_{21}-\lambda_{22}\right)V_{2}^{\frac{1}{2}}-\left(\bar{L}\lambda_{23}-\lambda_{24}\right)V_{2},$$

where $\lambda_{21}=\frac{\sqrt{\lambda_{\min}\left(P_{1}\right)}\lambda_{\min}\left(\Omega_{1}\right)}{\sqrt{2}\lambda_{\max}\left(P_{1}\right)}$, $\lambda_{22}=\gamma_{i2}\left\|\psi_{1}\right\|\sqrt{\frac{2}{\lambda_{\min}\left(P_{1}\right)}}$, $\lambda_{23}=\frac{2\lambda_{\min}\left(\Lambda_{1}\right)}{\lambda_{\max}\left(P_{1}\right)}$, and $\lambda_{24}=\frac{2\varsigma_{i}\left\|\Phi \right\|}{\lambda_{\min}\left(P_{1}\right)}$. □

**Remark** **6.**  *The actual control input* τ *is bounded because of the constraint (25). In practical application, the system should be controllable even if input saturation occurs. The desired control input* $\bar{\tau }$ *should ensure the stability of the closed-loop system, and be considered to be bounded. Otherwise, the designed control inputs* τ *and* $\bar{\tau }$ *is meaningless. The assumption has been used in* [37,38].

Similar to Theorem 1, when $\left|{\bar{s}}_{i}\right|>\sigma$ and $\bar{L}\geq L_{\min}$, the adaptive law becomes $\dot{\bar{L}}=r>0$. It means that the adaptive coefficient $\bar{L}$ starts to increase until the constraints $\bar{L}\lambda_{21}-\lambda_{22}>0$ and $\bar{L}\lambda_{23}-\lambda_{24}>0$ are met, which ensures the finite time stability of the dynamic system (33). Therefore, the sliding mode variable ${\bar{s}}_{i}$ always converges to a bounded region $\left|{\bar{s}}_{i}\right|\leq {\bar{\delta }}_{i1}$, where ${\bar{\delta }}_{i1}$ is a positive constant. Obviously, the auxiliary dynamic system (26) is uniformly ultimately bounded. So, the state χ is bounded. From Equation (27) and Remark 6, it is concluded that s always converges to a bounded region. And the error $e_{i}$ is driven into a bounded region. □

**Remark** **7**  **([30,32]).** *The generalized Lyapunov theorem only requires continuity and not differentiability of the Lyapunov function* $V_{2}$ *along the solution trajectories.* ${ℜ}_{2}^{\prime }=\left\{\left({\bar{s}}_{i},{\bar{s}}_{Ii}\right)\in R^{2n}:{\bar{s}}_{i}=0,{\bar{s}}_{Ii}=0\right\}$ *is an equilibrium of the differential Equation (33). From Equation (33), if* $\left({\bar{s}}_{i},{\bar{s}}_{Ii}\right)\in {ℜ}_{1}\backslash {ℜ}_{1}^{\prime }$*, then* ${\bar{s}}_{i}=0$ *and* ${\dot{\bar{s}}}_{i}={\bar{s}}_{Ii}\neq 0$*. Therefore, at least one component* ${\bar{s}}_{i}$ *will monotonically cross zero unless* $\left({\bar{s}}_{i},{\bar{s}}_{Ii}\right)$ *stay in the set* ${ℜ}_{2}^{\prime }$*.*

**Remark** **8.**  *When the designed control law (29) is not saturated (i.e., $\tilde{\tau }=0$), the auxiliary dynamic system (26) is asymptotically stable (i.e., χ→0 as t→∞). The designed controller (29) is almost the same as the controller (7) without considering saturation constraints. When the designed control law (29) is saturated (i.e., $\tilde{\tau }=0$), the state χ in the auxiliary dynamic system (26) varies with the change of $\tilde{\tau }$ and reduce the effect of the saturation constraint on the control performance.*

## 5. Simulations

In this section, a series of two-joint robot manipulators is used as an example. Its dynamic model is expressed as

(37) $$\left[\begin{array}{cc} M_{11} & M_{12}\\ M_{12} & M_{11} \end{array}\right]\left[\begin{array}{c} {\ddot{q}}_{1}\\ {\ddot{q}}_{2} \end{array}\right]+\left[\begin{array}{cc} -C_{11}{\dot{q}}_{1} & -2C_{11}{\dot{q}}_{1}\\ 0 & C_{11}{\dot{q}}_{2} \end{array}\right]\left[\begin{array}{c} {\dot{q}}_{1}\\ {\dot{q}}_{2} \end{array}\right]+\left[\begin{array}{c} G_{1}g\\ G_{2}g \end{array}\right]=\left[\begin{array}{c} \tau_{1}\\ \tau_{2} \end{array}\right]+\left[\begin{array}{c} \tau_{1d}\\ \tau_{2d} \end{array}\right],$$

where $M_{11}=\left(m_{1}+m_{2}\right)l_{1}^{2}+m_{2}l_{2}^{2}+2m_{2}l_{1}l_{2}\cos\left(q_{2}\right)+J_{1}$, $C_{11}=m_{2}l_{1}l_{2}\sin\left(q_{2}\right)$, $M_{12}=m_{2}l_{2}^{2}+m_{2}l_{1}l_{2}\cos\left(q_{2}\right)$, $G_{1}=\left(m_{1}+m_{2}\right)l_{1}\cos\left(q_{2}\right)+m_{2}l_{2}\cos\left(q_{1}+q_{2}\right)$, $M_{22}=m_{2}l_{2}^{2}+J_{2}$, and $G_{2}=m_{2}l_{2}\cos\left(q_{1}+q_{2}\right)$. The parameters are set to $m_{1}=0.5$ kg, $m_{2}=1.5$ kg, $l_{1}=1$ m, $l_{2}=0.8$ m, $J_{1}=5$ kg·m^2^ and $J_{2}=5$ kg·m^2^.

To confirm the performance of the presented technique, the following simulations are implemented to verify the state convergence on the SMS, robustness against uncertainties and disturbances, adaptive law, and control performance in the presence of input saturation, respectively.

### 5.1. Convergence of States on the Sliding Mode Surface

**Figure 2:**
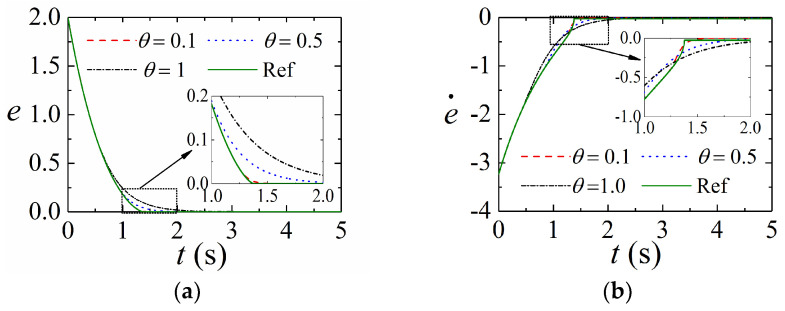
Convergence of states on the sliding mode surface: (**a**) The convergence process of tracking error; (**b**) The convergence process of the derivative of tracking error.

First, the convergence of states on the proposed SMS is verified. To this end, the fast terminal SMS $s=\dot{e}+c_{1}e+c_{2}e^{b_{1}/b_{2}}$ is chosen as a reference for comparison, where there are positive constants satisfying constraints $b_{2}>b_{1}$ and $b_{2}=2m+1$, m=1,2,3,⋯. The parameters are provided as $c_{1}=1$, $c_{2}=1$, $b_{1}=1$, $b_{2}=3$, and α=13. Let e(0)=2 as the initial state of the error e. When the SMS is reached, the dynamic convergence process of e and $\dot{e}$ is provided in Figure 2. From Figure 2, it shows that the two sliding-mode surfaces could be very close when the constant θ is small enough. It is recognized that there exists a singularity when State e equals to zero for the fast terminal SMS. In the proposed SMS, the function μ(e) is introduced to avoid this case. By choosing a very small value of θ, the proposed SMS is very close to the fast terminal SMS and does not cause the singular problem. Choosing a small value of θ is helpful to accelerate the convergence rate. In the actual implementation, the error may change around zero due to noise, friction, disturbance, and others, which could cause chattering. Choosing a large value of θ is helpful in suppressing chattering.

### 5.2. Robustness against Uncertainties and Disturbances

Next, to verify the advantage of the developed ASTSMC, comparisons are performed with the adaptive NFTSMC (ANFTSMC) in [26] and the adaptive nonlinear SMC (ANSMC) studied in [36]. ANFTSMC for the robotic system (37) is provided by

(38) $$\begin{array}{l} s=e+l_{1}{\mathrm{sig}}^{\beta_{1}}\left(e\right)+l_{2}{\mathrm{sig}}^{\beta_{2}}\left(\dot{e}\right)\\ \tau =\tau_{eq}+\tau_{asw}\\ \tau_{eq}=C_{0}\left(q,\dot{q}\right)\dot{q}+G_{0}\left(q\right)+M_{0}\left(q\right){\ddot{q}}_{d}-\frac{M_{0}\left(q\right)}{\beta_{2}l_{2}}{\left|\dot{e}\right|}^{2-\beta_{2}}\left(1+\beta_{1}l_{1}{\left|e\right|}^{\beta_{1}-1}\right)\mathrm{sign}\left(\dot{e}\right)\\ \tau_{asw}=M_{0}\left(q\right)\left[-hs-\left({\hat{b}}_{0}+{\hat{b}}_{1}\left\|q\right\|+{\hat{b}}_{2}{\left\|\dot{q}\right\|}^{2}\right)\mathrm{sign}\left(s\right)\right]\\ {\dot{\hat{b}}}_{0}=\lambda_{0}\left\|s\right\|{\left\|\dot{e}\right\|}^{\beta_{2}-1}\;\;\;\;\;{\dot{\hat{b}}}_{1}=\lambda_{1}\left\|s\right\|{\left\|\dot{e}\right\|}^{\beta_{2}-1}\left\|q\right\|\;\;\;\;\;{\dot{\hat{b}}}_{2}=\lambda_{2}\left\|s\right\|{\left\|\dot{e}\right\|}^{\beta_{2}-1}{\left\|\dot{q}\right\|}^{2} \end{array},$$

ANSMC for the robotic system (37) is provided as

(39) $$\begin{array}{l} s=G\left(\dot{e}+\rho e\right)\\ \tau =\tau_{0}^{\prime }+\tau_{1}^{\prime }+\tau_{2}^{\prime }\\ \tau_{0}^{\prime }=C_{0}\left(q,\dot{q}\right)\dot{q}+G_{0}\left(q\right)+M_{0}\left(q\right){\ddot{q}}_{d}\mathrm{sign}\left(s\right)\\ \tau_{1}^{\prime }=-M_{0}\left(q\right)\rho \dot{e}\\ \tau_{2}^{\prime }=M_{0}\left(q\right)G^{T}\left(-\varsigma_{1}s-\hat{\delta }\mathrm{sign}\left(s\right)\right)\\ \dot{\hat{\delta }}=\varsigma_{2}\left\|sG^{T}\right\| \end{array},$$

**Figure 3:**
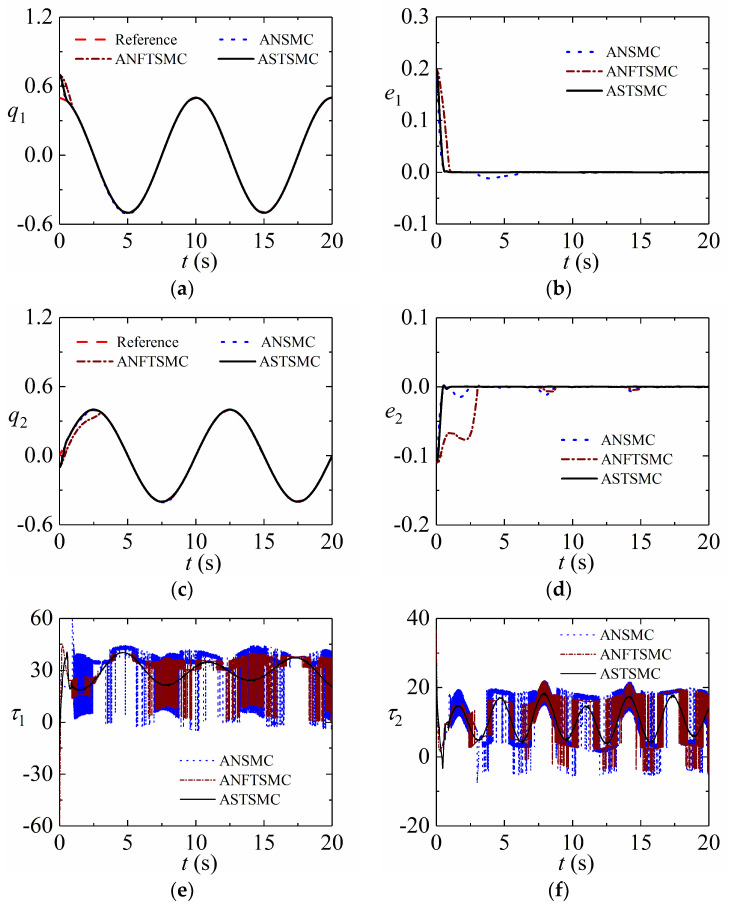
Tracking performance of robotic system with uncertainties and disturbances: (**a**) Position tracking of Joint 1; (**b**) Tracking error of Joint 1; (**c**) Position tracking of Joint 2; (**d**) Tracking error of Joint 2; (**e**) Control torque of Joint 1; (**f**) Control torque of Joint 2.

The nominal values of system parameters are set as $m_{10}=0.3$, $m_{20}=1$, $J_{10}=3$, $J_{20}=3$, $l_{10}=1.0$, and $l_{20}=0.8$. The initial states of the system are assumed as $q_{1}\left(0\right)=0.7$, $q_{2}\left(0\right)=-0.1$, ${\dot{q}}_{1}\left(0\right)=0$, and ${\dot{q}}_{2}\left(0\right)=0$. The disturbances and desired signals are provided by $\tau_{d1}=8\sin\left(t\right)+0.5\sin\left(200\pi t\right)$, $\tau_{d2}=6\cos\left(2t\right)+0.5\sin\left(200\pi t\right)$, $q_{d1}=0.5\cos\left(0.1\times 2\pi t\right)$, and $q_{d2}=0.4\sin\left(0.1\times 2\pi t\right)$. The gains of controllers are listed in Table 1. Figure 3 provides the responding simulation results in the presence of uncertainties and disturbances.

Figure 3a–d shows that the proposed ASTSMC achieves the highest tracking accuracy than others in the presence of uncertainties and disturbances. It indicates that the developed ASTSMC is robust to various disturbances. Figure 3e,f provides control inputs of these controllers. They have control inputs with the same magnitude. However, the control input of the proposed ASTSMC is smoothest, which indicates that the developed controller is chattering free.

### 5.3. Adaptive Law

To validate the superiority of the presented adaptive method, a recently presented adaptive law [29] is used for comparison. The adaptive law is provided as

(40) $${\dot{L}}^{\prime }=\{\begin{array}{ll} r & ,\left\|s\right\|>0\\ 0 & ,\left\|s\right\|=0 \end{array},$$

**Figure 4:**
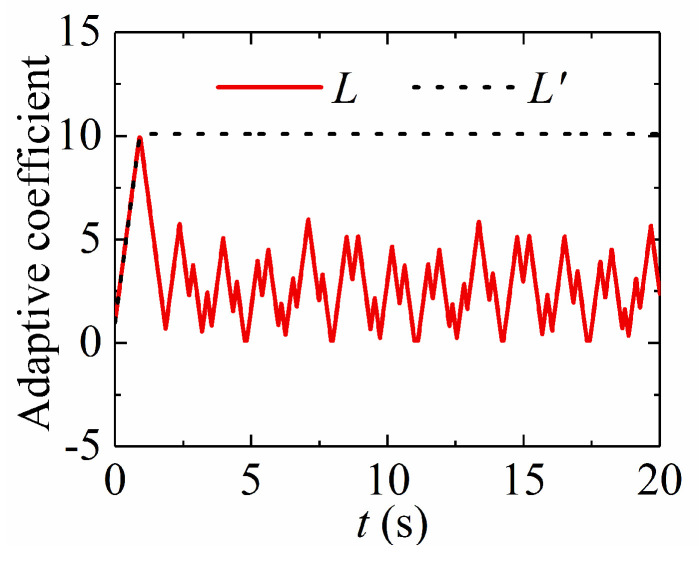
Adaptive process of adaptive coefficients.

**Figure 5:**
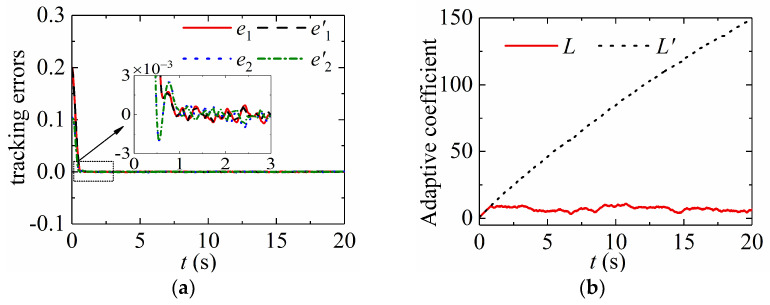
Simulation results with noise: (**a**) Tracking errors of Joint 1; (**b**) Adaptive coefficients.

**Figure 6:**
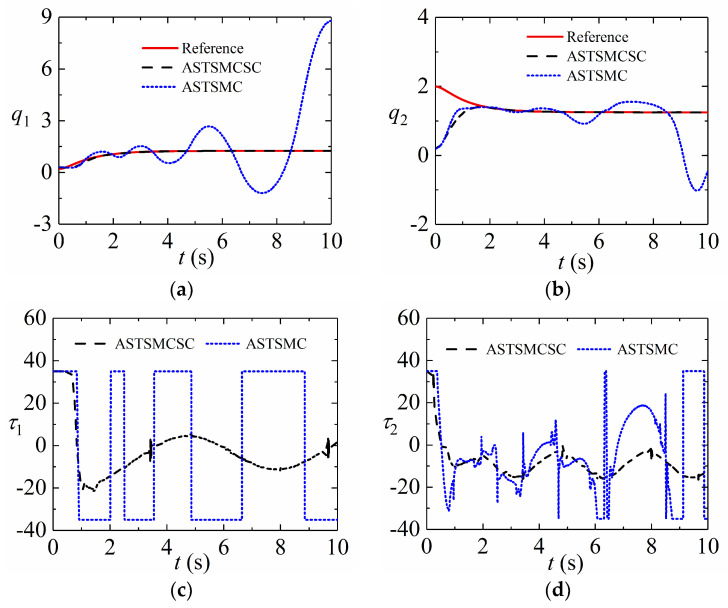
Simulation results in presence of input saturation: (**a**) Position tracking of Joint 1; (**b**) Position tracking of Joint 2; (**c**) Control torque of Joint 1; (**d**) Control torque of Joint 2.

Figure 4 provides the adaptive coefficients of the simulation in Section 5.2. Figure 5 shows that the presented adaptive law (8) can increase or decrease the control gains in terms of tracking errors. The adaptive law (40) increases the control gains all the time until the tracking error converges to zero and the adaptive coefficient stays at its maximum value. This case does not show the merits of the proposed method. A uniformly distributed stochastic noise signal is considered in the simulation. The feedback angular positions $q_{1}$ and $q_{2}$ are provided to the noise whose value is between −1.0 × 10^−3^ rad and +1.0 × 10^−3^ rad. Figure 6 presents the corresponding simulation results. Practically, tracking errors cannot converge zero due to violent noise, as shown in Figure 5a. Figure 5b confirms that the adaptive law (40) is increasing all the time since tracking errors are not zero, which makes the adaptive coefficient very large and decreases tracking performance. Thus, the proposed adaptive law has strong robustness and adaptability.

### 5.4. Control Performance in Presence of Input Saturation

In this section, the simulation will be implemented to further demonstrate ASTSMCSC. The initial values of states in Equation (37) are assumed as $q_{1}\left(0\right)=0.3$, $q_{2}\left(0\right)=0.2$, ${\dot{q}}_{1}\left(0\right)=0$ and ${\dot{q}}_{2}\left(0\right)=0$. The input saturation constraint is set as

(41) $$\tau =\{\begin{array}{ll} 35, & \mathrm{if}\;\bar{\tau }>35\;\\ \bar{\tau }, & \mathrm{if}\;-35\leq \bar{\tau }\leq 35\\ -35, & \mathrm{if}\;\bar{\tau }<-35 \end{array},$$

The control parameters of the ASTSMCSC (29) are the same as those of the ASTSMC (7). Other parameter is set as a=diag{5,5}. The desired signals are provided as $q_{d1}=1.25-\left(7/5\right)e^{-t}+\left(7/20\right)e^{-4t}$ and $q_{d2}=1.25+e^{-t}-\left(1/4\right)e^{-4t}$. Figure 6 presents the responding simulation results in the presence of the input saturation constraint. The control inputs are large since the initial states are far from the reference trajectories. As shown in Figure 6c,d, the control inputs are saturated at the beginning of the simulation. The proposed ASTSMCSC (29) adopts the saturation compensation to reduce the effect of input saturation constraint on tracking the performance of the system. It can be seen from Figure 6a,b that the proposed ASTSMCSC (29) could achieve a better tracking performance in the presence of input saturation constraint than the proposed ASTSMC. The control performance of ASTSMC becomes bad since the control inputs are saturated.

## 6. Conclusions

This paper investigates the trajectory control of series multiple-joint robot manipulators without input saturation and with input saturation. For robotic manipulators with uncertainties and disturbances, an ASTSMC is proposed, which improves tracking performance. In the presented control approach, a novel fast SMS is presented. It not only approximates the traditional fast terminal sliding surface infinitely but also avoids singularity problems. A modified adaptive law is introduced such that the disturbance/uncertainty with an unknown bounded derivative is handled by the proposed control approach. Considering input saturation in practical cases, an ASTSMCSC is proposed. When there is no input saturation, like the proposed control of robot manipulators without input saturation, it can achieve high tracking accuracy, strong robustness, and adaptability. When there exists input saturation, this control approach can reduce the effect of input saturation on the tracking performance of robot manipulators. A large number of simulations demonstrate the effectiveness of the theory and the developed approach.

## Figures and Tables

**Table 1:** Parameters of controllers.

Section	Parameters
ASTTSMC	α=0.5, $\theta_{1}=\theta_{2}=0.1$, $L_{0}=1$, κ=2, $L_{\min}=0.1$, r=10, $\hbar_{1}=\hbar_{2}=\mathrm{diag}\left\{1,1\right\}$, $k_{1}=k_{2}=k_{3}=k_{4}=\mathrm{diag}\left\{1,1\right\}$
ANFTSMC	$l_{1}=l_{2}=\mathrm{diag}\left\{0.1,0.1\right\}$, $\beta_{1}=2$, $\beta_{2}=1.5$, h=diag{5,5}, $\lambda_{0}=\lambda_{1}=50$, $\lambda_{2}=20$
ANSMC	ρ=diag{5,5}, G=diag{1,1}, $\varsigma_{1}=\mathrm{diag}\left\{20,20\right\}$, $\varsigma_{2}=\mathrm{diag}\left\{10,10\right\}$

## Data Availability

Data are contained within the article.
